# Potassium Humate and Plant Growth-Promoting Microbes Jointly Mitigate Water Deficit Stress in Soybean Cultivated in Salt-Affected Soil

**DOI:** 10.3390/plants11223016

**Published:** 2022-11-08

**Authors:** Khadiga Alharbi, Emadeldeen Rashwan, Emad Hafez, Alaa El-Dein Omara, Hossam Hussein Mohamed, Tarek Alshaal

**Affiliations:** 1 Department of Biology, College of Science, Princess Nourah bint Abdulrahman University, P.O. Box 84428, Riyadh 11671, Saudi Arabia; 2 Agronomy Department, Faculty of Agriculture, Tanta University, Tanta 31527, Egypt; 3 Department of Agronomy, Faculty of Agriculture, Kafrelsheikh University, Kafr El-Sheikh 33516, Egypt; 4 Agricultural Research Center, Microbiology, Soils, Water Environment Research Institute, Giza 12112, Egypt; 5 Department of Agronomy, Faculty of Agriculture, Ain Shams University, Cairo 13625, Egypt; 6 Soil and Water Department, Faculty of Agriculture, Kafrelsheikh University, Kafr El-Sheikh 33516, Egypt; 7 Department of Applied Plant Biology, Institute of Crop Sciences, University of Debrecen, Böszörményi Street 138, 4032 Debrecen, Hungary

**Keywords:** *Glycine max*, PGPMs, plant growth stimulants, oxidative stress, antioxidant enzymes, watering regimes, soil salinity

## Abstract

Lack of high-quality irrigation water and soil salinity are two main environmental factors that affect plant development. When both stressors are combined, the soil becomes sterile and constrains plant productivity. Consequently, two field trials were designed to assess whether plant growth-promoting microbes (PGPMs; *Bradyrhizobium japonicum* (USDA 110) and *Trichoderma harzianum*) and potassium humate (K-humate) can stimulate soybean growth, productivity, and seed quality under two different watering regimes as follows: (i) well-watered (WW), where plants were irrigated at 12-day intervals (recommended), and (ii) water stress (WS), where plants were irrigated at the 18-day intervals in salt-affected soil during 2020 and 2021 seasons. Results revealed that coupled application of PGPMs and K-humate resulted in a substantial improvement in K^+^ levels in the leaves compared to Na^+^ levels, which has a direct positive impact on an enhancement in the antioxidants defense system (CAT, POX, SOD), which caused the decline of the oxidative stress indicators (H_2_O_2_, MDA, and EL%) as well as proline content under water stress in salt-affected soil. Hence, a significant increase in root length, nodule weight, soybean relative water content (RWC), stomatal conductance, photosynthetic pigments, net photosynthetic rate, soluble protein, seed carbohydrate content as well as the number of pods plant^−1^ and seed yield was reported. In conclusion, the combined application of PGPMs and K-humate might be recommended to maximize the soybean growth and productivity under harsh growth conditions (e.g., water stress and soil salinity).

## 1. Introduction

Most lands dedicated to agricultural production face massive confronts such as salinity and water stress (drought or waterlogging), which eventually disturb plant development and decline in crop productivity and quality [1]. Some crops considerably adapt to such types of stressors with different degrees of response. Those crops cultivated in a tropical climate are often subjected to harmful abiotic stresses, including water deficit stress and high soil salinity [2]. It was reported that about 1.5 Mha of agricultural land is lost annually due to salinity [3]. Leguminous plants are moderately sensitive to salinity, which causes excessive ion toxicity (i.e., Na^+^ and Cl^−^) and osmosis disruption, leading to a declined potential of plant roots for water and nutrient uptake [4]. The excessive accumulation of these salts in plant tissues potentially injures plant tissues, resulting in tissue damage and cell death due to oxidative damage and osmotic stress [5]. One of the sustainable objectives to mitigate the damaging impacts of salinity is improving the osmoprotectants (e.g., proline, glycine betaine, and sugars), proteins, and different antioxidant enzymes (e.g., catalase (CAT), peroxidase (POD), and superoxide dismutase (SOD)) in the plant tissues, which have the potential to scavenge reactive oxygen species (ROS) [6].

Water deficit stress, one of the ecological stresses, is a limiting factor of plant development and crop productivity in most agricultural fields worldwide [7]. Increasing irrigation pump costs, insufficient irrigation system capacities, and reduced irrigation water provision are among the causes for many farmers intentionally using less water than is needed for maximum productivity [8]. The objective of effective management of irrigation water is to increase profitable yield with less use of water [9]. Different strategies have been implemented to reduce water consumption resulting in improved irrigation water shortage supplies while minimizing harmful impacts of water stress on yield [10]. Consequently, water deficit stress and soil salinity harm the physiological and biochemical processes in crops, resulting in low water potential and increased osmotic strain, and eventually reduced crop production [11].

Soybean (*Glycine max* L.) is the second most widely grown legume. Its seeds are rich in protein, carbohydrates, oil, and essential nutrients [12]. Soybean has been successfully cultivated in various soil types; however, a substantial decline in growth, development, and seed yield is documented, mainly due to soil salinity and water deficit stress [13]. Unfortunately, it is also among the most drought-susceptible crop plants. The situation worsens progressively in countries located in arid and semi-arid zones, such as Egypt. Similarly, soybean seeds are a good source of protein owing to their high content of indispensable amino acids [14]. On the other hand, soybean cultivation enhances soil fertility by adding substantial amounts of N into the soil matrix through N_2_-fixation by rhizobium bacteria, which colonize the root nodules [15].

Recently, the agricultural strategy has allowed several ways to maximize the growth and productivity of field crops, such as inoculating seeds with bacteria that live in the root zone of plants and interact with plants resulting in direct or indirect overgrowth [16]. These bacteria are known as plant growth-promoting microbes (PGPMs) due to their ability to increase the accessibility of microorganisms and nutrients to host plant roots. PGPMs are an efficient source of carbon inside the soil, which supports plants to grow well and combat abiotic stresses [17]. Rhizobacteria have the potential to increase water status and further increase water uptake under water deficit stress conditions [18]. Several mechanisms of PGPM action have been well documented, including siderophores production, biofilm, and plant hormones that stimulate plant growth [19]. Several plant hormones such as indole acetic acid, gibberellins, and other growth regulators produced by PGPMs could promote the physiological responses in the host plant and enhance root length and root surface area, resulting in improved nutrient uptake, thus counteracting salinity stress and inducing plant health under stress conditions [20]. Consequently, a successful strategy to mitigate the harmful impacts of soil salinity and water stress in plants could be the co-inoculation of seeds with various PGPM species [21]. Bradyrhizobium japonicum is a gram-negative bacterium with agronomical importance due to its high capability to colonize, specifically, soybean roots and symbiotically fix atmospheric N_2_ gas [22] (Isawa et al., 1999). Trichoderma sp. is well known as a plant biostimulant due to its high capacity to colonize plant root systems and help in supplying plants with nutrients in parallel with inducing plant resistance to several biotic and abiotic stresses such as plant diseases, salinity, and water deficit [23] (Harman, 2011) due to supporting root growth, facilitating nutrients uptake, and scavenging reactive oxygen species (ROS).

Another sustainable technology in crop production is foliar spraying, which transports metabolites in the phloem, supporting nutritional balance, increasing the organic compounds through photosynthesis and osmoregulation, and decreasing oxidative stress by improving the activity of antioxidant enzymes under stress conditions [24]. Soybean plants require a high fertilization rate of K [25]. Potassium is the most abundant cation in the cytoplasm [26]. Plants that accumulate substantial amounts of protein and carbohydrates inside their storage tissues have a high requirement for K. Potassium humate (K-humate) is commonly utilized as a source of K through foliar application [27]. Spraying plants with K-humate increases the permeability of plant membranes owing to humate application, which enhances root growth, increases cell division and improves productivity [28]. Humates are widespread carbonaceous materials of plant and animal residues resulting from biological and chemical decomposition [29]. K-humate is an effective natural material containing several macroelements such as N, P, and K and microelements such as Mo, Cu, Zn, B, Co, and Mg [30]. The accumulation of chlorophyll, sugar, and amino acids in plant tissues is a consequence of spraying plants with the K-humate, which enables the plants to tolerate the salt stress, eventually positively reflected on nutrient uptake, plant productivity, and quality as a result of a hormone-like effect and activation of photosynthesis [31]. These adaptive responses include tolerance of osmotic stress and exclusion of ions. K-humate is an inexpensive and environmentally friendly source of essential nutrients for the growth of the soybean plant [32].

There is a crucial requirement to examine eco-friendly sustainable applications, which possibly could enhance soybean production under water deficit stress in salt-affected soil. Therefore, aims of the present study were to (1) report the detrimental impacts of combined water deficit stress and salt salinity on soybean growth and productivity; (2) investigate the ameliorative potential of some PGPMs added singularly or jointly with K-humate on soybean development and yield; (3) study the improvement in antioxidant capacity of soybean under water deficit stress and soil salinity; and (4) monitor biochemical and physiological changes of soybean plants under water deficit stress in the presents of PGPMs and K-humate.

## 2. Results

### 2.1. Physiological Responses of Soybean to Plant Growth Stimulators under Water Deficit Stress

#### 2.1.1. Ion Accumulation

**Figure 1:**
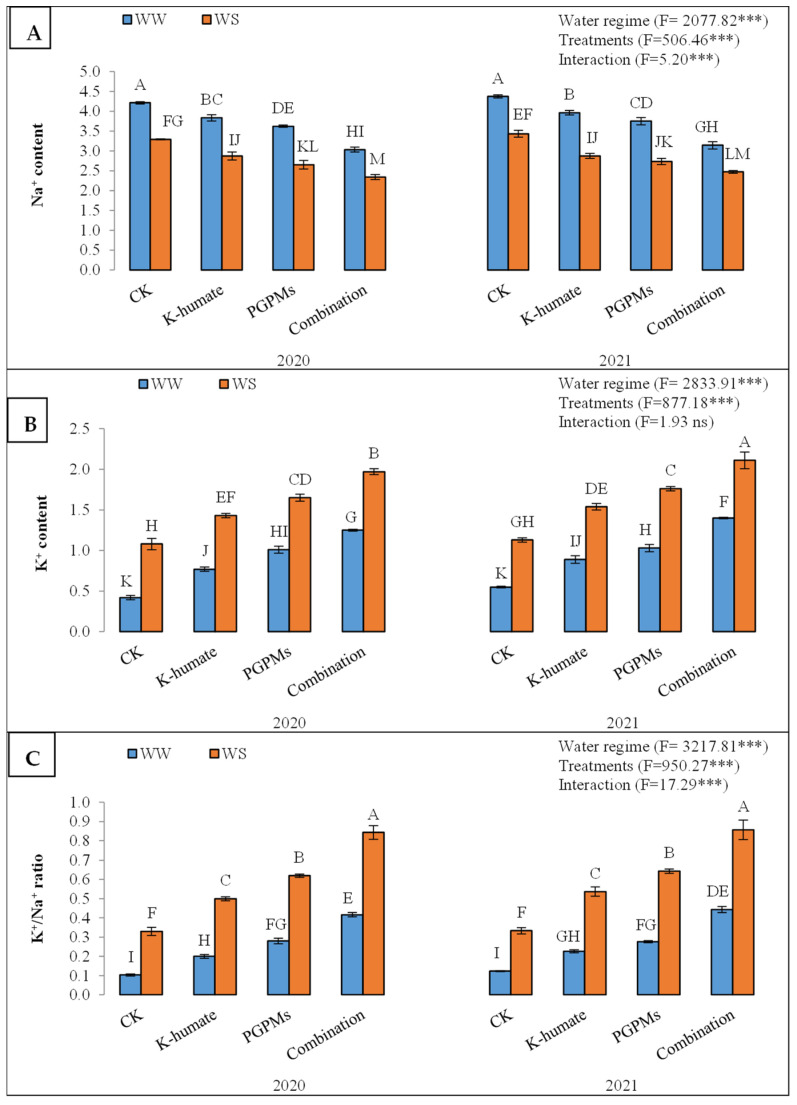
Uptake and accumulation of (**A**) Na^+^; (**B**) K^+^; and (**C**) K^+^/Na^+^ in soybean seeds (*Glycine max* L. cv. Giza 111) leaves, which was cultivated in salt-affected soil and treated with plant growth-promoting microbes (PGPMs, *Bradyrhizobium japonicum* (USDA 110), and *Trichoderma harzianum*), potassium humate (K-humate), and their combination under two water regimes (WS: water deficit regime and WW: well-watered regime) during two consecutive seasons. Different letters above columns show significant differences between treatments according to the Tukey’s test (*p* ≤ 0.05). Data are Means ± SD and n = 3. *** denotes significance at *p* ≤ 0.001, ns denotes insignificant difference.

The Na^+^ accumulation in leaf tissues was significantly higher under water deficit conditions (3.74%) than under well-watered conditions (2.83%). The K-humate and PGPMs significantly diminished the uptake and accumulation of Na^+^, recording lower Na^+^ contents than control. Yet, the highest reduction in the Na^+^ concentration corresponded to the treatment of K-humate + PGPMs, which recorded 2.75% (Figure 1). The treatment of K-humate + PGPMs exhibited the lowest Na^+^ concentration under both water regimes, followed by the single application of PGPMs and K-humate. The treatment of K-humate + PGPMs revealed a Na^+^ content of 3.03% and 3.14% under water deficit stress in 2020 and 2021, respectively, and 2.34% and 2.47% under well-watered conditions in 2020 and 2021, respectively.

The K^+^ content was significantly higher in the well-watered plants (1.58%) than in those which experienced water deficit stress (0.92%). In addition, the applied plant growth stimulators displayed higher K^+^ contents than control plants. The combined application of K-humate and PGPMs exhibited the highest K^+^ content (1.68%), followed by the PGPMs (1.36%) and K-humate (1.16%). The increase in K^+^ content upon treating plants with K-humate and PGPMs was significantly higher in plants that underwent the well-watered regime than those grown under water deficit conditions (Figure 1). Moreover, plants treated with the combined application of K-humate and PGPMs displayed the highest K^+^ content under both water regimes in the two years of the experiment.

Consequently, the K^+^/Na^+^ ratio was higher under well-watered conditions (0.58) than under water deficit stress (0.26). All evaluated plant growth stimulators showed a higher K^+^/Na^+^ ratio than control under both water regimes during the two years of the experiment. While the treatment of K-humate + PGPMs revealed the highest K^+^/Na^+^ ratio (0.64), the control plants had the lowest K^+^/Na^+^ ratio (0.22) (Figure 1). The same treatment (K-humate + PGPMs) resulted in the highest K^+^/Na^+^ ratio among all treatments. However, the increase in the K^+^/Na^+^ ratio under well-watered conditions due to the application of different plant growth stimulators was significantly higher than those under the water deficit stress.

#### 2.1.2. Efficiency of Soybean Photosynthetic Machinery under Different Water Regimes

**Figure 2:**
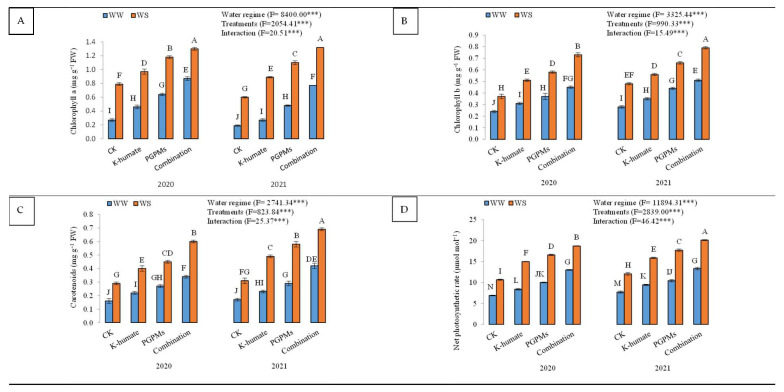
Changes in (**A**) chlorophyll a (chl a); (**B**) chlorophyll b (chl b); (**C**) carotenoids contents, and (**D**) net photosynthetic rate in soybean seeds (*Glycine max* L. cv. Giza 111) leaves, which were cultivated in salt-affected soil and treated with plant growth-promoting microbes (PGPMs; *Bradyrhizobium japonicum* (USDA 110) and *Trichoderma harzianum*), potassium humate (K-humate), and their combination under two water regimes (WS: water deficit regime and WW: well-watered regime) during two consecutive seasons. Different letters above columns show significant differences between treatments according to the Tukey’s test (*p* ≤ 0.05). Data are Means ± SD and n = 3. *** denotes significance at *p* ≤ 0.001.

The contents of photosynthetic pigments (chl a, chl b, and carotenoids) and net photosynthetic rate significantly lowered when soybean plants were exposed to water deficit conditions (Figure 2). Well-watered plants showed higher chl a (1.02 mg g^−1^ FW), chl b (0.59 mg g^−1^ FW), carotenoids (0.48 mg g^−1^ FW), and net photosynthetic rate (15.84 μmol m^−2^ s^−1^) than water-stressed plants. The single and combined application of K-humate and PGPMs revealed higher photosynthetic pigment contents and net photosynthetic rate than control under both water regimes during the whole period of the experiment. The treatment of K-humate + PGPMs resulted in the highest concentration of photosynthetic pigments and the highest rate of net photosynthesis, followed by the treatment of PGPMs and K-humate under both water regimes. The contents of chl a, chl b, and carotenoids were 0.87, 0.45, and 0.34 mg g^−1^ FW under water deficit conditions in 2020 and increased to 1.30, 0.73, and 0.60 mg g^−1^ FW under well-watered conditions of the same year. In addition, in 2020, the same treatment showed a net photosynthetic rate of 13.0 μmol m^−2^ s^−1^ and 18.7 μmol m^−2^ s^−1^ under water deficit and well-watered conditions, respectively. Similar results were reported in the second year.

#### 2.1.3. Generation of Oxidants under Water Deficit Stress

**Figure 3:**
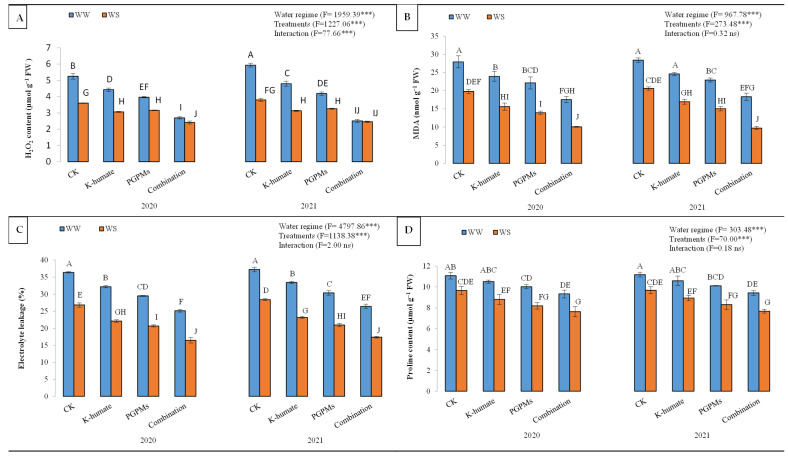
Variations in (**A**) H_2_O_2_ content; (**B**) malondialdehyde (MDA) content; (**C**) electrolyte leakage (EL), and (**D**) proline content in soybean seeds (*Glycine max* L. cv. Giza 111) leaves, which was cultivated in salt-affected soil and treated with plant growth-promoting microbes (PGPMs; *Bradyrhizobium japonicum* (USDA 110) and *Trichoderma harzianum*), potassium humate (K-humate), and their combination under two water regimes (WS: water deficit regime and WW: well-watered regime) during two consecutive seasons. Different letters above columns show significant differences between treatments according to the Tukey’s test (*p* ≤ 0.05). Data are Means ± SD and n = 3. *** denotes significance at *p* ≤ 0.001, ns denotes insignificant difference.

The production of H_2_O_2_ in soybean plants under water deficit stress increased significantly compared to those grown under well-watered conditions. However, K-humate and PGPMs significantly diminished the generation of H_2_O_2_ in cells of soybean, where they recorded lower H_2_O_2_ contents than the control. The H_2_O_2_ content in control plants was 4.65 µmol g^−1^ FW and reduced to 2.52 µmol g^−1^ FW upon treating plants with K-humate + PGPMs (Figure 3). Although the single application of K-humate or PGPMs significantly suppressed the generation of H_2_O_2_, the highest reduction in the H_2_O_2_ content corresponded to the treatment of K-humate + PGPMs, regardless of the water regime. For instance, in 2020, H_2_O_2_ concentration reduced from 5.25 µmol g^−1^ FW at control to 2.70 µmol g^−1^ FW at K-humate + PGPMs under water deficit stress. Yet, lower concentrations of H_2_O_2_ were reported under well-watered conditions.

Oxidation of bilayer in plasma membrane significantly increased under water deficit conditions (Figure 3). The malondialdehyde (MDA) content, as an indicator of lipid peroxidation, increased from 15.20 nmol g^−1^ FW (under well-watered conditions) to 23.23 nmol g^−1^ FW (under water deficit stress). The exogenous application of K-humate and PGPMs significantly reduced the MDA content. The highest reduction in MDA content corresponded to the treatment of K-humate + PGPMs, followed by PGPMs and K-humate. Control plants had an MDA content of 24.18 nmol g^−1^ FW, while plants treated with K-humate + PGPMs displayed an MDA content of 13.90 nmol g^−1^ FW. The combined application of K-humate and PGPMs revealed the lowest MDA content under both water regimes during the two years of the experiment. For example, in 2020, the MDA reduced from 27.93 (for control) to 17.53 (for K-humate + PGPMs treatment) nmol g^−1^ FW under water deficit stress, while it declined from 19.80 (for control) to 10.03 (for K-humate + PGPMs treatment) nmol g^−1^ FW under the well-watered conditions.

Electrolyte leakage (EL) shows the damage degree to plant tissues under abiotic stress. Plants exposed to water deficit stress showed a significant and higher EL value (31.33%) than well-watered plants (22.01%). Plants that received K-humate and PGPMs displayed lower EL values than the control. Overall, EL values were 32.23% (for control), 27.73% (for K-humate), 25.38% (for PGPMs), and 21.36% (for K-humate + PGPMs). The treatment of K-humate + PGPMs exhibited the lowest EL values under both water regimes; however, plants grown under the well-watered regime showed lower EL values under all studied plant growth stimulators. Treating soybean with K-humate + PGPMs resulted in the lowest EL values under both water regimes during 2020 and 2021.

Plants that grew under water deficit stress showed higher proline content (10.28 μmol g^−1^ FW) than those grown under well-watered conditions (8.62 μmol g^−1^ FW). The treatment of K-humate + PGPMs displayed the lowest proline content (8.52 μmol g^−1^ FW), followed by PGPMs (9.16 μmol g^−1^ FW) and K-humate (9.71 μmol g^−1^ FW), while the control had a proline content of 10.40 μmol g^−1^ FW. The combined application of K-humate and PGPMs revealed the lowest proline content under both water regimes during the two years of the experiment.

#### 2.1.4. Activities of Antioxidant Enzymes in Soybean under Different Water Regimes

**Figure 4:**
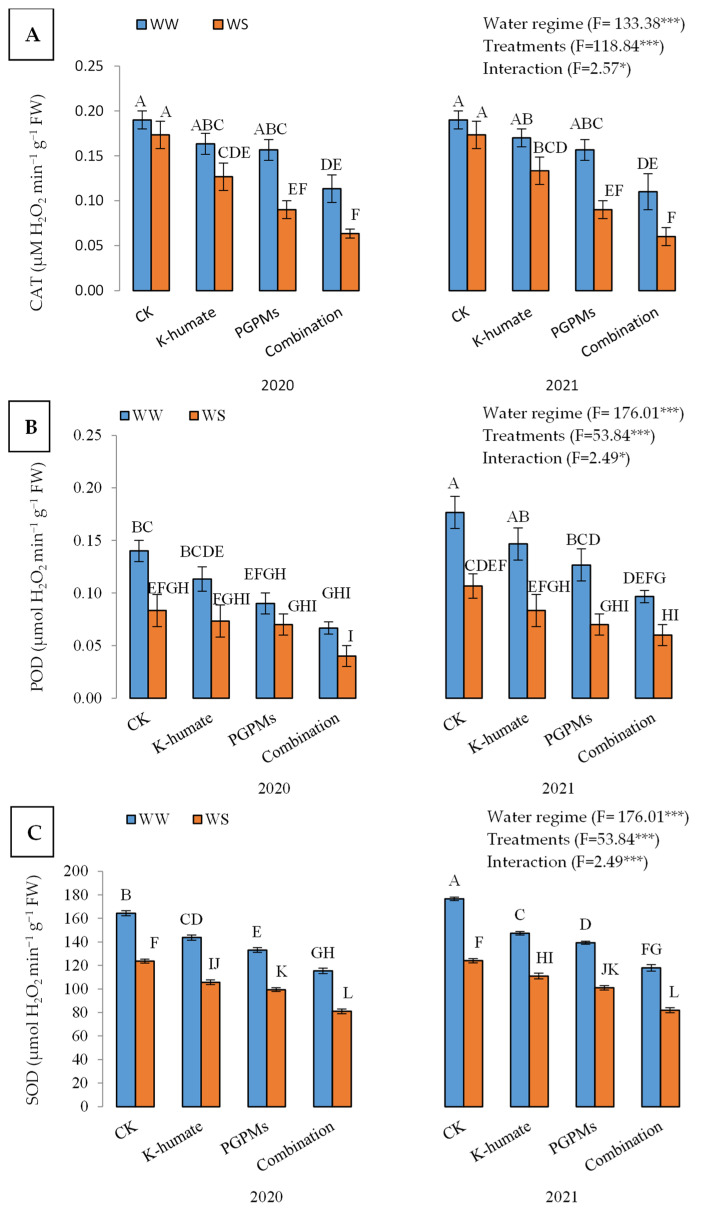
Activities of different antioxidant enzymes: (**A**) catalase (CAT); (**B**) peroxidase (POD), and (**C**) superoxide dismutase (SOD) in soybean seeds (*Glycine max* L. cv. Giza 111) leaves, which was cultivated in salt-affected soil and treated with plant growth-promoting microbes (PGPMs; *Bradyrhizobium japonicum* (USDA 110) and *Trichoderma harzianum*), potassium humate (K-humate), and their combination under two water regimes (WS: water deficit regime and WW: well-watered regime) during two consecutive seasons. Different letters above columns show significant differences between treatments according to the Tukey’s test (*p* ≤ 0.05). Data are Means ± SD and n = 3. *** denotes significance at *p* ≤ 0.001, * denotes significance at *p* ≤ 0.05.

Higher activities of antioxidant enzymes, i.e., catalase (CAT), peroxidase (POD), and superoxide dismutase (SOD) were measured in leaves of soybean plants exposed to water deficit stress than those that followed the well-watered regime (Figure 4). Under the water deficit regime, activities of CAT (µM H_2_O_2_ min^−1^ g^−1^ FW), POD (µmol H_2_O_2_ min^−1^ g^−1^ FW), and SOD (µmol H_2_O_2_ min^−1^ g^−1^ FW) were 0.156, 0.120, and 142, respectively, and dropped down to 0.114, 0.073, and 103, respectively, under the well-watered regime. The application of K-humate and PGPMs resulted in lower activities of antioxidant enzymes compared to control plants. Under the treatment of K-humate + PGPMs, the activities of CAT, POD, and SOD were 0.087, 0.066, and 99, respectively, while control plants showed activities of 0.182, 0.127, and 147, respectively, regardless of the water regime. The lowest activities of the three enzymes under water deficit and well-watered conditions in both years corresponded to the treatment of K-humate + PGPMs. The treatment of K-humate + PGPMs displayed activities of 0.11, 0.067, and 115 of CAT, POD, and SOD under water deficit conditions in 2020, while control showed activities of 0.19, 0.140, and 164 of CAT, POD, and SOD, respectively. However, under well-watered conditions, the activities of CAT, POD, and SOD were lower.

### 2.2. Alterations in Soybean Biometrics and Biochemical Composition under Water Deficit Stress

#### 2.2.1. Plant Biometrics

Soybean plants exposed to water deficit stress possessed a significantly shorter root system (20.0 cm) than well-watered plants (26.1 cm). All treatments displayed a taller root system of soybean plants than control, regardless of the water regime. The tallest root system (27.1 cm) corresponded to the combined application of K-humate + PGPMs, followed by the single application of K-humate (24.1 cm) and PGPMs (22.1 cm), while control plants had a root length of 19.0 cm (Table 1). The exogenous application of K-humate and PGPMs enhanced the soybean growth under the water deficit and well-watered conditions in both seasons; however, the well-watered plants possessed taller root systems. The K-humate + PGPMs treatment showed the tallest root system compared to the other treatments, regardless of the water regime. In 2020, the root length increased from 22.5 cm to 30.5 cm after treating plants with the K-humate + PGPMs under the water deficit and well-watered conditions, respectively.

The dry mass of nodules significantly reduced upon allowing soybean plants to grow under water deficit conditions recording 261 mg plant^−1^, while the dry mass of nodules of the well-watered plants was 309 mg plant^−1^. Plants treated with K-humate and PGPMSs showed higher nodule dry mass than control. The highest nodule dry mass (311 mg plant^−1^) corresponded to the treatment of K-humate + PGPMs. The single or combined addition of K-humate and PGPMs alleviated the detrimental impacts of the water deficit stress. The treatment of K-humate + PGPMs resulted in the highest nodule dry mass under the water deficit and well-watered conditions during both seasons, recording 285 and 331 mg kg^−1^ (for 2020) and 290 and 338 mg kg^−1^ (for 2021), respectively.

Water-stressed plants showed lower stomatal conductance (35.3) than well-watered ones (42.2). In addition, plants treated with K-humate and PGPMs displayed higher stomatal conductance than control plants. The highest stomatal conductance (43.1) corresponded to the combined application of K-humate and PGPMs, while the control showed the lowest stomatal conductance (34.6). Under water deficit stress, stomatal conductance was significantly reduced; however, the applied substances mitigated the harmful effects of water deficit by increasing the stomatal conductance. The combined application of K-humate and PGPMs resulted in the highest increase in the stomatal conductance of soybean plants under water deficit and well-watered conditions during the two years of the experiment (Table 1).

The relative water content (RWC) significantly decreased when soybean plants grown in salt-affected soil were exposed to water deficit (71.3%) compared to well-watered ones (79.5%). Control plants showed the lowest RWC (70.3%), while plants treated with K-humate + PGPMs displayed the highest RWC (81.0%), regardless of the applied water regime (Table 1). Plants treated with K-humate and/or PGPMs showed higher RWC than control under both water regimes; however, higher values were documented in the case of well-watered plants. The combined application of K-humate and PGPMs showed the highest RWC under water deficit (75.8% and 77.0% in 2020 and 2021, respectively) and well-watered (85.2% and 85.8% in 2020 and 2021, respectively) conditions.

#### 2.2.2. Biochemical Components in Soybean Leaves Grown under Water Deficit Stress

The content of total soluble protein in soybean leaves significantly declined under water deficit conditions (9.62 mg g^−1^ FW) compared to the well-watered regime (15.80 mg g^−1^ FW) (Table 2). Both K-humate (11.65 mg g^−1^ FW) and PGPMs (13.74 mg g^−1^ FW) applications showed higher total soluble protein content than the control (9.16 mg g^−1^ FW); moreover, the combined K-humate + PGPMs application revealed the highest concentration (16.30 mg g^−1^ FW). Regardless of the water regime, the applied substances (K-humate and PGPMs) resulted in higher total soluble protein contents than the control. For instance, in 2020, the combined application of K-humate and PGPMs showed the highest total soluble protein content under water deficit (12.11 mg g^−1^ FW) and well-watered (19.39 mg g^−1^ FW) conditions. Similar findings were reported in the second year.

In contrast, the content of free amino acids in soybean leaves significantly increased under water deficit conditions (30.59 mg g^−1^ FW) compared to the well-watered regime (23.46 mg g^−1^ FW). Leaves of control plants showed the highest free amino acids content (31.28 mg g^−1^ FW) compared to plants treated with K-humate and PGPMs, regardless of the water regime (Table 2). The treatment of K-humate + PGPMs revealed the lowest free amino acids content (22.19 mg g^−1^ FW). The same treatment displayed the lowest concentration of free amino acids under both water regimes during the two years of the experiment, recording 25.6 and 26.6 mg g^−1^ FW under the water deficit regime in 2020 and 2021, respectively, and 17.8 and 18.7 mg g^−1^ FW under the well-water management in 2020 and 2021, respectively.

### 2.3. Soybean Seed Yield and Nutritional Quality under Water Deficit Stress

#### 2.3.1. Seed Yield-Related Traits

Shortage supply of irrigation water (water deficit stress) significantly lowered the soybean yield as it diminished the number of pods (Table 3). The number of pods decreased from 96.3 pods per plant (for well-watered plants) to 81.4 pods per plant (for water-stressed plants). The number of pods per plant significantly differed according to the applied treatments. For instance, the combined application of K-humate and PGPMs displayed the highest number of pods (99.3 pods per plant) compared to their single applications (91.8 and 85.8 pods per plant for K-humate and PGPMs, respectively) and control (78.3 pods per plant). Although water deficit lowered the number of pods, the single and combined application of K-humate and PGPMs significantly improved soybean productivity by increasing the number of pods per plant. However, the highest number of pods of plants exposed to water deficit conditions corresponded to the combined application of K-humate and PGPMs in both growing seasons, recording 86.6 pods per plant (for 2020) and 90.7 pods per plant (for 2021). A higher increase in the number of pods upon the application of K-humate + PGPMs was recorded for well-watered plants.

Soybean plants grown under water deficit conditions displayed a lower 100-seed weight (16.2 g) than the well-watered plants (18.0 g). The exogenous application of K-humate and PGPMs improved the 100-seed weight compared to control; yet, the highest 100-seed weight (18.2 g) corresponded to the combined application of K-humate + PGPMs, followed by the single application of PGPMs (17.4 g) and K-humate (16.8 g). The single and combined application of K-humate and PGPMs induced the 100-seed weight of soybean under water deficit and well-watered conditions. However, higher values were recorded in the case of well-watered than water deficit conditions. The combined application of K-humate and PGPMs exhibited the highest 100-seed weight under both water regimes in both growing seasons, followed by the single application of the PGPMs and K-humate (Table 3).

The well-watered plants showed a higher seed yield (1.99 ton ha^−1^) than the water-stressed ones (1.72 ton ha^−1^) (Table 3). The K-humate and PGPMs significantly increased the soybean seed yield compared to the control. The highest seed yield (2.02 ton ha^−1^) corresponded to the treatment of K-humate + PGPMs, followed by the PGPMs (1.89 ton ha^−1^) and K-humate (1.80 ton ha^−1^). The well-watered plants showed a higher seed yield than the water-stressed plants; however, the K-humate and PGPMs ameliorated the seed yield under both water regimes. The highest seed yield corresponded to the combined application of K-humate and PGPMs under water deficit and well-watered conditions.

#### 2.3.2. Nutritional Quality of Soybean Seeds under Water Stress Conditions

Seed protein content significantly differed in response to water regimes and applied treatments (Table 3). Well-watered plants showed significantly higher seed protein content (22.01%) than those grown under water deficit conditions, recording 13.91%. In addition, the K-humate and PGPMs markedly influenced the seed protein content compared to the control. The seed protein content increased from 13.18% (for control) to 22.58% (for the K-humate + PGPMs). The applied K-humate and PGPMs significantly enhanced the seed protein content of soybean plants grown under the two water regimes; nevertheless, well-watered plants showed higher seed protein content. The combined application of K-humate and PGPMs exhibited the highest seed protein content among all treatments under water deficit (17.96 and 19.10% in 2020 and 2021, respectively) and well-watered conditions (26.48 and 26.78% in 2020 and 2021, respectively).

Similarly, the carbohydrate content of soybean seed varied significantly due to the applied water regime in the presence of K-humate and PGPMs (Table 3). Water deficit stress considerably reduced the seed carbohydrate content compared to well-watered plants. The seed carbohydrate content increased from 11.9% (for control) to 17.4% (for the K-humate + PGPMs treatment). In addition, PGPMs showed higher seed carbohydrate content (15.3%) than K-humate (14.1%). Water deficit significantly lessened the seed carbohydrate content, yet the application of K-humate and PGPMs in singular or combined form mitigated the detrimental impacts of water stress. Furthermore, the application of K-humate and PGPMs enhanced the seed carbohydrate content in seeds of well-watered plants. The combined application of K-humate and PGPMs resulted in the highest seed carbohydrate content under both water regimes during the two growing seasons.

### 2.4. Correlation Matrix between Soybean Parameters

Pearson correlation matrix (2-tailed) showed significant correlation between most soybean growth and yield parameters (Table S1). Water regime displayed positive, very strong, and significant (*p* < 0.01) correlation with root length, nodule mass, chl a, chl b, net photosynthetic rate, RWC, stomatal conductance, K^+^ content, K^+^/Na^+^ ratio, seed yield, seed protein and carbohydrate contents, and contents of leaf soluble protein and leaf total soluble sugar. It also showed positive, strong, and significant (*p* < 0.01) correlation with carotenoids content, H_2_O_2_ content, number of pods, and weight of 100-seed. On the other hand, the Na^+^ content, SOD activity, MDA content, EL, and leaf free amino acid quantity revealed negative, very strong, and significant (*p* < 0.01) with water regime. A negative, strong, and significant (*p* < 0.01) correlation was reported between water regime and activities of CAT and POD.

## 3. Discussion

Organoregulation is believed to be one of the key necessary strategies in plant adaptation to combat water stress and soil salinity [31,32,33]. It can lessen the osmotic capacity of plant cytosol into the vacuole due to the accumulation of low molecular weight organic solutes and inorganic ions, consequently increasing water-holding capacity and necessary nutrient (active removal of Na^+^) in the soil solution, all of which improve soil chemical and physical properties as well as positively affect water and nutrient uptake [34]. The present investigation has been carried out to assess the response of soil salinity and water stress as a consequence of sole and combination application of PGPMs as seed inoculation and foliar spraying with K-humate on alleviation of the negative impacts of oxidative damage as well as improving antioxidant enzymes activity, resulting in improvement of physiological processes and soybean productivity under stressors [35]. The findings illustrated above obviously designate that PGPMs and/or potassium humate tested herein contributed to the augment in the tolerance of the soybean plants to water stress and soil salinity as evidenced by our measurements [36]. The Na^+^ or Cl^−^ ions are considered to be the main factors of these nutrition problems which constrain nutrient availability [37]. A reduction in the soil exchangeable Na^+^ with an increase in K^+^ in soil solution was observed as a result of application of PGPMs (*B. japonicum* and *T. harzianum*), which resulted in improved soil physical and chemical properties [38]. Application of PGPM (*B. japonicum* and *T. harzianum*) strains could produce phytohormones (IAA, GA3, and zeatin), which has the potential for symbiotic N_2_ fixation due to nodule formation and root development as a consequent of increased number of lateral roots and root hairs as well as the nodules number [39]. In addition, these microbial strains could colonize soybean roots, which resulted in significant augments in root length and nodules, reflecting positively on growth attributes, physiological processes and eventually seed yield [39]. Application of K-humate as foliar spraying declined soil exchangeable Na^+^ and positively reflected on nodule weight and root length [40]. The observed improvement is related to the positive role of K which plays a key role in the biosynthesis of auxins and endogenous GA3 [41], and consequently enhances root length and nodules due to an active role in cell enlargement [42]. Furthermore, humic acid has a lot of valuable impacts on plant quality [42]. Humic acid application to leguminous crops had the potential to augment N_2_-fixing rhizobacteria, increasing root length and number of nodules [43]. It was observed that combined application of PGPMs and K-humate significantly declined soil exchangeable Na^+^ by 32.5% compared to control; however, nodule dry weight augmented by 19.1% and root length by 28.6% over control [43]. These promising results are in conformity with our prior study on sugar beet, where the combined application of PGPMs and K-humate decreased soil exchangeable Na^+^ and improved the soil properties [44].

The chl a content decreased significantly under water stress in saline soil; the same trend was found in chl b and carotenoids [45]. The chl a, b and carotenoids were found to be the highest due to the combined application of PGPMs and K-humate under well-watered treatment in saline soil [45]. The noticeable improvement in photosynthetic pigments under water stress in saline soils may be due to an increase in the transporter of K^+^ from the soil solution to plant cells in leaves and a decrease in the uptake of Na^+^ ions [46], which positively induces enzymes responsible for synthesis of the physiological processes such as the net rate of photosynthesis, stomatal conductance, and RWC. These findings also confirm those reported in other studies [47].

Higher net photosynthetic rate, stomatal conductance, and RWC in soybean plants subjected to water stress and grown in saline soil were noticed due to increased CO_2_ influx, transpiration rate, and photosystem II which, induced by combined application of PGPMs and K-humate, resulted in the production of plant growth hormones and IAA synthesis that increased water uptake together with increased nutrient uptake by vacuoles, collecting mainly in the cytosol [48].

It was demonstrated in our investigation that combined application of PGPMs with K-humate had higher positive effect on osmolytes, photosynthetic pigments, oxidative phosphorylation, protein polymerization enzymatic activities, and physiological processes than sole application under soil salinity which accelerated mineralization and augmented availability of nutrients. These findings are in agreement with those noted by [49,50]. Activity of antioxidant enzymes such as CAT, SOD, and POD were increased in soybean plants, causing a reduction in Na^+^ uptake and detoxification of the negative effects of reactive oxygen species (ROS) [51]. Increased activity of antioxidant enzymes upon the combined application of PGPMs and K-humate could convert H_2_O_2_ into non-toxic compounds (H_2_O and O_2_), therefore protecting plants from the adverse effects of drought and salinity on cell membranes and macromolecules under salt-affected soil as well as the decline in MDA compared to control treatment. These factors would finally lead to a highly beneficial effect on physiological attributes and productivity under soil salinity [52]. Not only oxidative damage indicators but also EL and proline content decreased as a result of increased cell water potential, osmoregulation and affecting hydraulic conductivity in the cytoplasm of plants [53]. Combined application of PGPMs and K-humate resulted in augmented stomatal index of the soybean plants, which increased the production of growth hormones necessary for chlorophyll formation and pollen grain formation that eventually positively reflected on number of pods plant^−1^ and 100-seed weight as well as enhanced metabolic processes in plant tissues [54], carbohydrate and protein synthesis alongside their transport to the developed seed. The subsequent showed high panicle fertility with low sterility coupled with heavy panicle caused high yield under soil salinity [55]. Combined application of PGPMs with K-humate is considered as a beneficial mineral nutrient for the reproductive growth and harvest index under soil salinity in addition to increase grain N, P and K whilst decreased Na^+^ [56]. As seen in the present study, the usage of PGPMs with K-humate could be a beneficial approach to addressing the growing dilemma of soil salinity, resulting in nutritional balance in plants and decreasing specific ionic toxicity such Na^+^ as well as increasing the growth and productivity of soybean [5,14].

## 4. Materials and Methods

***Experimental location and sources of used materials***

Open field experiments were conducted at the Elamaar village, Sidi Salem, Kafr El-sheik Governorate, Egypt (31°07′ N, 30°57′ E) during summer seasons (May to September) of 2020 and 2021. The region where the experiment was established is characterized by the following meteorological parameters (on average): minimum air temperature of 17.58 °C, maximum air temperature of 32.55 °C, wind speed 100.42 (km day^−1^), relative humidity of 66.65%, and precipitation of 0% within the period between May and September. Soybean seeds (*Glycine max* L. cv. Giza 111) were kindly attained from the Field Crops Research Institute, Department of Leguminous Crops, Agricultural Research Station, Sakha, Egypt. The two PGPM strains (*Bradyrhizobium japonicum* (USDA 110) and *Trichoderma harzianum*) were obtained from the Bacteriology Laboratory, Sakha Agricultural Research Station, Kafr El-Sheikh, Egypt as peat-based inoculums. Potassium humate (K-humate; humic acid 85%, K_2_O 8%, and fulvic acid 3%) was purchased from the Egyptian Company for Fertilizers and Chemicals, Cairo, Egypt.

***Experimental layout***

The experiments were set up in a Randomized Complete Block Design (RCBD) with the factorial arrangement in a strip plot with four repeats. Soybean plants grown in salt-affected soil were exposed to two different watering regimes as follows: (i) well-watered (WW), where plants were irrigated at 12-day intervals (recommended), and (ii) water stress (WS), where plants were irrigated at the 18-day intervals. The basic physicochemical traits of the surface layer (0–30 cm) of the experimental soil are presented in Table 4.

To alleviate the detrimental impacts of water stress and soil salinity, soybean plants were treated consortium of PGPMs, K-humate, and their combination, while control plot (CK) did not receive PGPMs or K-humate. Sub-plot area was 42 m^2^ including 10 rows, 7 m long × 6 m wide. Soybean seeds were sown at a rate of 100 kg seed ha^−1^ on 15 May 2020 and 21 May 2021 in one side of ridges. Soybean seeds were inoculated with PGPMs strains in a shadow directly before seed sowing at a rate of 950 g ha^−1^, where 100 mL (1 × 10^8^ CFU mL^−1^) of each PGPMs strain was thoroughly mixed with a 200 g carrier using a sticking material. The K-humate was applied on the foliage part of soybean plants at a rate of 450 L ha^−1^ (according to the manufacture’s recommendations) and it was sprayed thrice at 40, 55, and 70 days after seed sowing.

The recommended doses of N, P, and K of soybean (according to the recommendations of the Ministry of Agriculture and Land Reclamation, Egypt) were 50, 80, and 114 kg ha^−1^, respectively, added as urea (46.5% N), calcium superphosphate (15.5% P_2_O_5_), and potassium sulfate (48% K_2_O). However, nitrogen fertilization was added as urea (46.5% N) at the rate of 18 kg N ha^−1^ with the treatment of seed inoculation with PGPMs. The other agronomic practices such as weeding and controlling pests and insects were done according to the recommendations of the Ministry of Agriculture and Land Reclamation, Egypt.

***Record of plant biometrics***

At 80 days after seed sowing, five plants were randomly selected to assess the root length (cm) and nodules dry mass (mg plant^−1^). The collected roots were oven-dried at 65 °C until the weight became constant. Nodules were carefully separated from the roots and oven-dried to obtain their dry mass after thoroughly washing with distilled water.

***Determination of K^+^ and Na^+^ content in the leaves***

First five fully expanded leaves from the plant tip were randomly gathered at 80 days after seed sowing from each treatment, and then washed with deionized water. The fresh leaves were oven-dried at 65 °C for 72 h until the weight became constant. A 200 mg dried sample was placed into 250 mL Kjeldahl tube and digested by a mixture of acids HNO_3_:HClO_4_ (2:1 *v/v*) for 120 min at 220 °C. The contents of Na^+^ and K^+^ in the digested samples were quantified using the Atomic Absorption Spectrophotometer (AAS, PERKIN ELMER 3300) with a detection limit of 100 ppb according to method of Chapman and Pratt [57]. The K^+^/Na^+^ ratio was calculated based on the obtained results of K^+^ and Na^+^.

***Determination of chlorophyll (a and b) and carotenoid contents***

Quantification of chl a, chl b, and total carotenoids was carried out at 80 days after seed sowing using the first five fully expanded leaves from the plant tip according to Lichtenthaler [58]. Briefly, 100 mg leaf tissues were crushed and extracted in 5 mL 80% acetone. The supernatants were collected by centrifugation for 10 min at 13,000 rpm. The absorbance of supernatant was read at 663 nm (for chl a), 645 nm (for chl b), and 470 nm (for total carotenoids). The concentrations (mg g^−1^ FW) of chl a, chl b, and total carotenoids were calculated using the following formulas:

chl a = 12.7 (A663) − 2.69 (A645),

chl b = 25.8 (A645) − 4.68 (A663).

Total carotenoids = [1000 (A470) − 2.27 (chl a) − 81.4 (chl b)]/227.

***Oxidative stress indicators***

The contents of H_2_O_2_ and malondialdehyde (MDA) in soybean leaves were detected at 80 days after seed sowing in the uppermost fully expanded leaves. A 500 mg fresh tissue was ground and homogenized in the present of liquid nitrogen and trichloroacetic acid (0.1%). The H_2_O_2_ content was measured in the supernatant collected by centrifugation for 20 min at 3000 rpm. The absorbance was recorded at 390 nm by a spectrophotometer (the model UV-160A spectrophotometer, Shimadzu, Japan) and the H_2_O_2_ content was expressed as µmol g^−1^ FW as stated by Velikova et al. [59]. The content of MDA (as an indicator of lipid peroxidation of cell membrane) was quantified as stated by Du and Bramlage [60]. Briefly, hydro-acetone buffer (4:1 *v/v*) 0.65% thiobarbituric acid (TBA) and 0.01% butyl hydroxyl toluene (BHT) were added and samples were incubated for 10 min at 95 °C in water bath. Afterward, the supernatants were collected by centrifugation at 10,000 rpm for 15 min. The absorbance was measured spectrophotometrically (The model UV-160A spectrophotometer, Shimadzu, Japan) at 532 and 600 nm and MDA concentration was expressed as nmol g^−1^ FW.

***Determination of activity of antioxidant enzymes (CAT, POD and SOD)***

The first five fully expanded leaves from the plant tip and terminal buds were detached at 80 days after seed sowing to assess the activity of catalase (CAT), peroxidase (POD), and superoxide dismutase (SOD). A 500 mg fresh leaf tissue was crushed and homogenized in 3 mL TRIS buffer (50 mM, pH 7.8), including 1 mM EDTA-Na. The activity of CAT (µM H_2_O_2_ min^−1^ g^−1^ FW) was estimated based on Aebi [61], while POD activity D (µmol H_2_O_2_ min^−1^ g^−1^ FW) was measured using the technique stated by Beauchamp and Fridovich [62]. The SOD activity (µmol H_2_O min^−1^ g^−1^ FW) was determined according to the method stated by Vetter et al. [63]. The model UV-160A spectrophotometer, Shimadzu, Japan was applied in measuring the activities of antioxidant enzymes.

***Measurements of physiological traits***

***Net photosynthetic rate (P_n_)***

The first five fully expanded leaves from the plant tip and terminal buds were detached at 80 days after seed sowing to assess the net photosynthetic rate. It was measured in the fine-days irradiance of 1000 μmol m^−2^ s^−1^ with a *LI-6400* portable photosynthesis Device (*Li-COR*, Lincoln, NE, USA) under temperature of 30 ± 2 °C, CO_2_ 350 to 400 μmol mol^−1^ and VPD of 50% RH.

***Stomatal conductance (g_s_)***

The first five fully expanded leaves from the plant tip and terminal buds were detached at 80 days after seed sowing to measure stomatal conductance. It was assessed in the fine days using the leaf porometer apparatus (Model AP4, Delta-T apparatus Ltd., Cambridge, UK). The following is the equation in the leaf in front (r_a_) and back surface (r_b_):

Stomatal conductance (r_l_) is 1∕r_l_ = 1∕r_a_ + 1∕r_b_.

***Leaf relative water content (RWC)***

The first five fully expanded leaves from the plant tip and terminal buds were detached at 80 days after seed sowing to evaluate RWC. It was assessed via the technique of Weatherley [64]. Biological replicates obtained were weighted to measure the fresh weight, and therefore rehydrated in distilled water and were put in the dark for a day and weighted again to obtain the turgid weight. Finally, the dry weight was obtained when leaves were oven-dried at 60 °C for 48 h. LRWC was computed using the following formula:

$$\mathrm{RWC}=\frac{\left(\mathrm{fresh}\;\mathrm{mass}+\;\mathrm{dry}\;\mathrm{mass}\right)}{\left(\mathrm{turgid}\;\mathrm{mass}-\mathrm{dry}\;\mathrm{mass}\right)}\times 100.$$

***Electrolyte leakage (EL)***

The first five fully expanded leaves from the plant tip and terminal buds were detached at 80 days after seed sowing to assess EL. It was assessed due to the technique stated by Bajji et al. [65]. Ten leaf discs were placed in a testing tube containing 10 mL distilled water, and the electrical conductivity (EC_1_) was calculated. The contents were then heated to 45–55 °C for 30 min each in a water bath and the electrical conductivity (EC_2_) was computed. The sample was oven-dried at 100 °C for 10 min, and the electrical conductivity (EC_3_) was calculated. The total leakage of inorganic ions was measured using the following formula:

$$\mathrm{EL}\;\left(\%\right)=\frac{\left(\mathrm{EC}2-\;\mathrm{EC}1\right)}{\left(\mathrm{EC}3\right)}\times 100.$$

***Osmo-protectants and protein concentrations***

***Free proline content (Pr)***

The first five fully expanded leaves from the plant tip and terminal buds were collected at 80 days after seed sowing to determine the free proline content (μmol g^−1^ FW leaves) using the methods described by Bates et al. [66]. A 500 mg fresh leaf tissue was ground with 3% aqueous sulfosalicylic acid and centrifuged at 11,500 rpm at 4 °C for 15 min. A 2 mL supernatant was obtained and 2 mL of glacial acetic acid and acid ninhydrin reagent was added. The reaction mixture was heated in water bath for 60 min, followed by quick cooling using ice. Next, 4 mL of toluene was applied and samples were incubated at room temperature for half hour. The absorbance of colored toluene was read at 520 nm using the model UV-160A spectrophotometer, Shimadzu, Japan.

Pro (μmol g^−1^ FW) = k value × dilution factor × absorbance/fresh sample weight.

where K value = 17.52, dilution factor = 2, fresh sample weight = 0.5 g.

***Free amino acid (FAA)***

Five fully expanded leaves from the plant tip and terminal buds were randomly collected at 80 days after seed sowing to measure the free amino acid content as described by Misra et al. [67]. Shortly, 500 mg fresh leaf tissue was extracted with 80% ethanol and centrifuged at 8000× *g* for 15 min at 4 °C. One milliliter of ninhydrin reagent (16% ninhydrin dissolved in citrate buffer, pH 5.0 [26% citric acid, and 58% Na citrate]) was added to supernatant and then incubated in water bath for 10 min. After cooling, absorbance of the reaction was measured at 570 nm using spectrophotomer (the model UV-160A spectrophotometer, Shimadzu, Japan). The standard curve was generated using L-Leucine.

***Total Soluble Protein (TSP)***

Five fully expanded leaves from the plant tip and terminal buds were randomly collected at 80 days after seed sowing to assess the concentration of soluble protein (mg g^−1^ FW) according to the method stated by Bradford [68] using the Brilliant Blue G-250 reagent with bovine serum albumin (BSA) as a standard.

***Total soluble sugar (TSS)***

Five fully expanded leaves from the plant tip and terminal buds were randomly collected at 80 days after seed sowing to measure the total soluble sugar (mg g^−1^ FW) using the method of Hendrix [69] by spectrophotometer (The model UV-160A spectrophotometer, Shimadzu, Japan) at 620 nm wavelength according to glucose standard curve. Biological replicates (0.5 g) were combined in 5 mL of 80% (*v/v*) ethanol and put in a water bath at 80 °C for 30 min, and then centrifuged at 10,000× *g* for 10 min.

***Seed yield related traits and nutritional quality***

At harvest time, the soybean plants of each treatment were harvested. Ten plants were used for computing the number of pods per plants. Dry soybean seeds were detached from their pods to measure 100-seed weight and seed soybean yield ha^−1^. Seed protein (%) and carbohydrate contents (%) were analyzed as described by AOAC (Association of official agriculture chemists) as stated by A.O.A.C. [70] and colorimetrically by the phenol-sulphuric acid technique as stated by Sadasivam and Manickam [71], respectively.

***Statistical analysis***

Normality of dependent variables was tested and converted as necessary. Data analysis was performed using Microsoft Excel 2016 and the SPSS 25.0 software package (SPSS Inc., Chicago, IL, USA). The analysis of variance using one-way ANOVA was performed separately between treatments, seasons, or water regimes. Separation of means was performed by post hoc test (Tukey’s test), and significant differences were accepted at the level *p* ≤ 0.05. The analysis of variance using two-way ANOVA was performed between water regimes, seasons, and treatments. The data were presented as mean ± standard deviation.

## 5. Conclusions

It is summarized that the exposure of soybean plants to water stress simultaneously with soil salinity resulted in oxidative damage and nutritional discharge which negatively affected plant growth and productivity. Consequently, the coupled application of seed inoculation and PGPMs (*Bradyrhizobium japonicum* (USDA 110) and *Trichoderma harzianum*) and foliar application with potassium humate alleviated the harmful impacts of water deficit and soil salinity on soybean growth, resulting in the highest seed quality (i.e., protein and carbohydrate content) and highest yield-related traits (number of pods plant^−1^ and 100-seed weight) of soybean plants. Multiple benefits for agricultural sustainability are associated with the coupled application of PGPMs and K-humate, especially in harsh environmental zones. However, future research experiments are required to affirm the attained findings on a large scale.

## Figures and Tables

**Table 1:** Some physical features of soybean plants (*Glycine max* L. cv. Giza 111) which were cultivated in salt-affected soil and treated with plant growth-promoting microbes (PGPMs; *Bradyrhizobium japonicum* (USDA 110) and *Trichoderma harzianum*), potassium humate (K-humate), and their combination under two water regimes (WS: water deficit regime and WW: well-watered regime) during two consecutive seasons.

			Root Length (cm)	Nodules Dry Mass (mg Plant^−1^)	Stomatal Conductance (mmol m^−2^ s^−1^)	RWC ^†^ (%)
**Water regime**						
WS			20.0 ± 0.11 b	261 ± 2.50 b	35.5 ± 0.47 b	71.3 ± 0.18 b
WW			26.1 ± 0.36 a	309 ± 1.90 a	42.2 ± 0.60 a	79.5 ± 0.37 a
**Treatments**						
Control			19.0 ± 0.42 d	257 ± 1.31 d	34.6 ± 0.75 d	70.3 ± 0.88 d
K-humate			24.1 ± 0.21 b	295 ± 1.01 b	37.9 ± 0.19 c	74.1 ± 0.49 c
PGPMs			22.1 ± 0.33 c	279 ± 2.74 c	39.6 ± 0.46 b	76.3 ± 0.52 b
Combination			27.1 ± 0.16 a	311 ± 3.00 a	43.1 ± 0.21 a	81.0 ± 0.38 a
**Interaction**						
2020	WS	Control	15.9 ± 0.10 g	236 ± 3.52 g	30.9 ± 0.23 h	65.6 ± 0.35 g
		K-humate	20.4 ± 0.28 e	268 ± 2.47 e	33.9 ± 0.40 g	69.4 ± 0.89 f
		PGPMs	18.4 ± 0.11 f	253 ± 2.50 f	35.7 ± 0.47 f	71.4 ± 0.18 e
		Combination	22.5 ± 0.36 d	285 ± 1.90 d	39.4 ± 0.60 d	75.8 ± 0.37 c
	WW	Control	21.0 ± 0.29 e	275 ± 1.41 e	37.1 ± 0.32 e	73.9 ± 0.62 d
		K-humate	26.5 ± 0.2 b	318 ± 2.34 b	40.9 ± 0.03 c	77.4 ± 0.83 c
		PGPMs	24.4 ± 0.37 c	305 ± 4.32 c	42.7 ± 0.37 b	79.9 ± 0.85 b
		Combination	30.5 ± 0.39 a	331 ± 2.48 a	45.6 ± 0.30 a	85.2 ± 0.65 a
2021	WS	Control	17.2 ± 0.42 g	237 ± 1.31 g	32.5 ± 0.75 h	67.3 ± 0.88 g
		K-humate	21.8 ± 0.21 e	269 ± 1.01 e	34.9 ± 0.19 g	70.9 ± 0.49 f
		PGPMs	19.7 ± 0.33 f	252 ± 2.74 f	36.5 ± 0.46 f	72.9 ± 0.52 e
		Combination	24.0 ± 0.16 d	290 ± 3.00 d	40.1 ± 0.21 d	77.0 ± 0.38 c
	WW	Control	21.7 ± 0.26 e	279 ± 1.03 e	38.0 ± 0.18 e	74.2 ± 0.55 d
		K-humate	27.5 ± 0.44 b	324 ± 1.00 b	41.9 ± 0.29 c	78.7 ± 0.46 c
		PGPMs	25.6 ± 0.22 c	305 ± 2.11 c	43.7 ± 0.32 b	80.9 ± 0.48 b
		Combination	31.4 ± 0.03 a	338 ± 0.82 a	47.3 ± 0.19 a	85.8 ± 0.45 a
**Two-way ANOVA (F-value)**						
Water regime			5356.73 ***	5123.95 ***	3841.98 ***	2285.00 ***
Treatments			1697.19 ***	1196.70 ***	1079.61 ***	673.70 ***
Interaction			16.46 ***	7.07 ***	3.27 *	1.72 ns

Means in the same column followed by the different letters are significant according to the Tukey’s test (*p* ≤ 0.05). Data are Means ± SD and n = 3. *** denotes significance at *p* ≤ 0.001, * denotes significance at *p* ≤ 0.05, ns denotes insignificant difference. ^†^ Relative water content.

**Table 2:** Some biochemical features of soybean leaves (*Glycine max* L. cv. Giza 111) which were cultivated in salt-affected soil and treated with plant growth-promoting microbes (PGPMs; *Bradyrhizobium japonicum* (USDA 110) and *Trichoderma harzianum*), potassium humate (K-humate), and their combination under two water regimes (WS: water deficit regime and WW: well-watered regime) during two consecutive seasons.

			Leaves (mg g^−1^ FW)		
			Total Soluble Protein	Free Amino Acids	Total Soluble Sugars
**Water regime**					
WS			9.62 ± 0.06 b	30.59 ± 0.81 a	1.11 ± 0.09 b
WW			15.80 ± 0.20 a	23.46 ± 0.48 b	2.10 ± 0.05 a
**Treatments**					
Control			9.16 ± 0.43 d	31.28 ± 0.09 a	1.07 ± 0.09 d
K-humate			11.65 ± 0.28 c	28.28 ± 0.50 b	1.41 ± 0.07 c
PGPMs			13.74 ± 0.34 b	26.34 ± 0.34 c	1.67 ± 0.04 b
Combination			16.30 ± 0.33 a	22.19 ± 0.37 d	2.28 ± 0.13 a
**Interaction**					
2020	WS	Control	6.61 ± 0.35 h	33.7 ± 0.26 a	0.72 ± 0.07 g
		K-humate	7.76 ± 0.43 g	31.4 ± 0.25 b	0.97 ± 0.09 fg
		PGPMs	9.81 ± 0.06 f	29.2 ± 0.81 c	1.14 ± 0.09 ef
		Combination	12.11 ± 0.20 d	25.6 ± 0.48 e	1.56 ± 0.05 cd
	WW	Control	10.60 ± 0.10 e	27.2 ± 0.40 d	1.40 ± 0.07 de
		K-humate	14.40 ± 0.10 c	24.4 ± 0.44 f	1.80 ± 0.20 c
		PGPMs	16.59 ± 0.06 b	22.2 ± 0.35 g	2.12 ± 0.05 b
		Combination	19.39 ± 0.38 a	17.8 ± 0.15 h	2.91 ± 0.11 a
2021	WS	Control	7.71 ± 0.43 h	35.5 ± 0.09 a	0.70 ± 0.09 g
		K-humate	8.92 ± 0.28 g	32.2 ± 0.50 b	0.99 ± 0.07 fg
		PGPMs	10.85 ± 0.34 f	30.4 ± 0.34 c	1.21 ± 0.04 ef
		Combination	13.21 ± 0.33 d	26.6 ± 0.37 e	1.62 ± 0.13 cd
	WW	Control	11.71 ± 0.05 e	28.6 ± 0.41 d	1.45 ± 0.12 de
		K-humate	15.51 ± 0.46 c	25.1 ± 0.24 f	1.88 ± 0.17 c
		PGPMs	17.73 ± 0.18 b	23.6 ± 0.44 g	2.20 ± 0.21 b
		Combination	20.48 ± 0.45 a	18.7 ± 0.31 h	3.01 ± 0.07 a
**Two-way ANOVA (F-value)**					
Water regime			5040.51 ***	3843.99 ***	907.31 ***
Treatments			1218.88 ***	1097.77 ***	244.33 ***
Interaction			21.76 ***	2.30 *	5.68 ***

Means in the same column followed by the different letters are significant according to the Tukey’s test (*p* ≤ 0.05). Data are Means ± SD and n = 3. *** denotes significance at *p* ≤ 0.001, * denotes significance at *p* ≤ 0.05, ns denotes insignificant difference.

**Table 3:** Yield and yield-related traits of soybean seeds (*Glycine max* L. cv. Giza 111) which were cultivated in salt-affected soil and treated with plant growth-promoting microbes (PGPMs; *Bradyrhizobium japonicum* (USDA 110) and *Trichoderma harzianum*), potassium humate (K-humate), and their combination under two water regimes (WS: water deficit regime and WW: well-watered regime) during two consecutive seasons.

			# Pods	100-Seed Weight (g)	Seed Yield (ton ha^−1^)	Seed Protein (%)	Seed Carbohydrate (%)
**Water regime**							
WS			81.4 ± 0.46 b	16.2 ± 0.41 b	1.72 ± 0.02 b	13.91 ± 0.31 b	12.2 ± 0.53 b
WW			96.3 ± 0.95 a	18.0 ± 0.12 a	1.99 ± 0.01 a	22.01 ± 0.04 a	17.2 ± 0.46 a
**Treatments**							
Control			78.3 ± 0.56 d	15.9 ± 0.01 d	1.71 ± 0.02 d	13.18 ± 0.19 d	11.9 ± 0.11 d
K-humate			85.8 ± 0.24 c	16.8 ± 0.32 c	1.80 ± 0.01 c	16.91 ± 0.45 c	14.1 ± 0.23 c
PGPMs			91.8 ± 0.43 b	17.4 ± 0.46 b	1.89 ± 0.09 b	19.16 ± 0.24 b	15.3 ± 0.11 b
Combination			99.3 ± 0.19 a	18.2 ± 0.07 a	2.02 ± 0.02 a	22.58 ± 0.05 a	17.4 ± 0.17 a
**Interaction**							
2020	WS	Control	71.3 ± 0.52 h	14.5 ± 0.48 f	1.55 ± 0.03 d	9.72 ± 0.04 h	9.0 ± 0.25 h
		K-humate	78.8 ± 0.48 g	15.0 ± 0.08 ef	1.64 ± 0.02 d	11.98 ± 0.19 g	10.7 ± 0.09 g
		PGPMs	82.2 ± 0.46 e	15.7 ± 0.41 de	1.76 ± 0.02 c	13.66 ± 0.31 f	12.0 ± 0.53 f
		Combination	86.6 ± 0.95 d	16.3 ± 0.12 cd	1.84 ± 0.01 c	17.96 ± 0.04 d	14.6 ± 0.46 d
	WW	Control	80.4 ± 0.56 f	16.1 ± 0.01 d	1.81 ± 0.02 c	15.97 ± 0.19 e	13.5 ± 0.11 e
		K-humate	89.8 ± 0.24 c	17.0 ± 0.32 bc	1.84 ± 0.01 bc	20.67 ± 0.45 c	16.3 ± 0.23 c
		PGPMs	98.0 ± 0.43 b	17.6 ± 0.46 b	1.94 ± 0.09 b	23.07 ± 0.24 b	17.5 ± 0.11 b
		Combination	106.5 ± 0.19 a	18.9 ± 0.07 a	2.08 ± 0.02 a	26.48 ± 0.05 a	19.6 ± 0.17 a
2021	WS	Control	76.2 ± 0.11 h	15.6 ± 0.43 f	1.61 ± 0.04 d	10.22 ± 0.44 h	10.5 ± 0.54 h
		K-humate	80.4 ± 0.43 g	16.8 ± 0.14 ef	1.69 ± 0.04 d	13.00 ± 0.13 g	12.1 ± 0.47 g
		PGPMs	84.7 ± 0.92 e	17.2 ± 0.26 de	1.77 ± 0.03 c	15.62 ± 0.42 f	13.0 ± 0.40 f
		Combination	90.7 ± 0.29 d	18.1 ± 0.05 cd	1.92 ± 0.02 c	19.10 ± 0.60 d	15.3 ± 0.22 d
	WW	Control	85.4 ± 0.85 f	17.6 ± 0.47 d	1.87 ± 0.08 c	16.82 ± 0.48 e	14.6 ± 0.42 e
		K-humate	94.4 ± 0.77 c	18.2 ± 0.24 bc	2.01 ± 0.05 bc	22.01 ± 0.33 c	17.3 ± 0.27 c
		PGPMs	102.4 ± 0.38 b	18.9 ± 0.16 b	2.11 ± 0.06 b	24.29 ± 0.24 b	18.4 ± 0.30 b
		Combination	113.4 ± 1.00 a	19.5 ± 0.17 a	2.24 ± 0.05 a	26.78 ± 0.45 a	20.0 ± 0.07 a
**Two-way (F-value)**							
Water regime			7451.57 ***	456.92 ***	474.03 ***	7089.50 ***	2757.70 ***
Treatments			2663.32 ***	130.90 ***	118.00 ***	1680.90 ***	571.63 ***
Interaction			77.42 ***	1.90 ns	2.26 *	13.91 ***	3.55 **

Means in the same column followed by the different letters are significant according to the Tukey’s test (*p* ≤ 0.05). Data are Means ± SD and n = 3. *** denotes significance at *p* ≤ 0.001, ** denotes significance at *p* ≤ 0.01, * denotes significance at *p* ≤ 0.05, ns denotes insignificant difference.

**Table 4:** Physicochemical traits of the surface layer (0–30 cm) of the experimental soil during the 2020 and 2021 seasons.

Season	Soil Texture	pH ^¥^	EC ^†^ (dSm^−1^)	FC ^‡^ (%)	SOM * (%)	Cations (meq L^−1^)				Anions (meq L^−1^)		
						Na^+^	K^+^	Mg^2+^	Ca^2+^	Cl^−^	HCO_3_^−^	SO_4_^2−^
2020	Clay loam	8.22 ± 0.01 a	6.88 ± 0.05 a	28.75 ± 0.09 a	1.45 ± 0.01 a	16.74 ± 0.03 b	9.36 ± 0.01 b	12.35 ± 0.08 b	18.63 ± 0.06 a	22.36 ± 0.04 b	14.36 ± 0.02 b	19.66 ± 0.05 b
2021	Clay loam	8.17 ± 0.02 b	6.76 ± 0.04 b	29.36 ± 0.11 a	1.39 ± 0.03 b	17.63 ± 0.05 a	10.24 ± 0.02 a	17.26 ± 0.06 a	17.35 ± 0.08 b	25.36 ± 0.04 a	17.54 ± 0.02 a	22.25 ± 0.03 a

^¥^ soil pH (measured in a soil:distilled water suspension (1:2.5)). ^†^ EC = Electric conductivity (measured in a soil paste extract). ^‡^ FC = Field capacity. * SOM = Soil organic matter.

## Data Availability

All data are available within the text.

## Supplementary Materials

The following supporting information can be downloaded at: https://www.mdpi.com/article/10.3390/plants11223016/s1.

## References

[1] Osman H.S., Gowayed S.M., Elbagory M., Omara A.E.-D., El-Monem A.M.A., Abd El-Razek U.A., Hafez E.M. (2021). Interactive Impacts of Beneficial Microbes and Si-Zn Nanocomposite on Growth and Productivity of Soybean Subjected to Water Deficit under Salt-Affected Soil Conditions. Plants.

[2] Hafez E., Farig M. (2019). Efficacy of salicylic acid as a cofactor for ameliorating effects of water stress and enhancing wheat yield and water use efficiency in saline soil. Int. J. Plant Prod..

[3] Alharbi K., Rashwan E., Mohamed H.H., Awadalla A., Omara A.E.-D., Hafez E.M., Alshaal T. (2022). Application of Silica Nanoparticles in Combination with Two Bacterial Strains Improves the Growth, Antioxidant Capacity and Production of Barley Irrigated with Saline Water in Salt-Affected Soil. Plants.

[4] Kheir A.M.S., Abouelsoud H.M., Hafez E.M., Ali O.A.M. (2019). Integrated effect of nano-Zn, nano-Si, and drainage using crop straw–filled ditches on saline sodic soil properties and rice productivity. Arab. J. Geosci..

[5] Omara A.E.D., Hafez E.M., Osman H.S., Rashwan E., El-Said M.A., Alharbi K., Abd El-Moneim D., Gowayed S.M. (2022). Collaborative Impact of Compost and Beneficial Rhizobacteria on Soil Properties, Physiological Attributes, and Productivity of Wheat Subjected to Deficit Irrigation in Salt Affected Soil. Plants.

[6] El-Shamy M.A., Alshaal T., Mohamed H.H., Rady A.M., Hafez E.M., Alsohim A.S., Abd El-Moneim D. (2022). Quinoa Response to Application of Phosphogypsum and Plant growth promoting microbesunder Water Stress Associated with Salt-Affected Soil. Plants.

[7] Hafez E.M., Osman H.S., Gowayed S.M., Okasha S.A., Omara A.E., Sami R., El-Monem A.M.A., El-Razek U.A.A. (2021). Minimizing the adversely impacts of water deficit and soil salinity on maize growth and productivity in response to the application of plant growth promoting microbesand silica nanoparticles. Agronomy.

[8] Seleiman M.F., Hafez E.M., Awaad H., Abu-hashim M., Negm A. (2021). Optimizing Inputs Management for Sustainable Agricultural Development. Mitigating Environmental Stresses for Agricultural Sustainability in Egypt.

[9] El-Saadony F.M.A., Mazrou Y.S.A., Khalaf A.E.A., El-Sherif A.M.A., Osman H.S., Hafez E.M., Eid M.A.M. (2021). Utilization Efficiency of Growth Regulators in Wheat under Drought Stress and Sandy Soil Conditions. Agronomy.

[10] Abdelrasheed K.G., Mazrou Y., Omara A.E.-D., Osman H.S., Nehela Y., Hafez E.M., Rady A.M.S., El-Moneim D.A., Alowaiesh B.F., Gowayed S.M. (2021). Soil Amendment Using Biochar and Application of K-Humate Enhance the Growth, Productivity, and Nutritional Value of Onion (*Allium cepa* L.) under Deficit Irrigation Conditions. Plants.

[11] Osman H.S., Rady A.M.S., Awadalla A., Omara A.E.-D., Hafez E.M. (2022). Improving the antioxidants system, growth, and sugar beet quality subjected to long-term osmotic stress by phosphate solubilizing bacteria and compost tea. Inter. J. Plant Prod..

[12] Moretti L.G., Crusciol C.A., Kuramae E.E., Bossolani J.W., Moreira A., Costa N.R., Alves C.J., Pascoaloto I.M., Rondina A.B.L., Hungria M. (2020). Effects of growth-promoting bacteria on soybean root activity, plant development and yield. Agron. J..

[13] Umezawa T., Shimuzu K., Kato M., Ueda T. (2000). Enhancement of salt tolerance in soybean with NaCl pretreatment. Physiol. Plant..

[14] Phang T.H., Shao G.H., Lam H.M. (2008). Salt tolerance in soybean. J. Integr. Plant Biol..

[15] Singleton P.W., Bohlool B.B. (1984). Effect of salinity on nodule formation by soybean. Plant Physiol..

[16] Hafez E.M., Gharib H.S. (2016). Effect of foliar application of ascorbic acid on physiological and biochemical characteristics of wheat under water stress. Inter. J. Plant Prod..

[17] Nehela Y., Mazrou Y.S.A., Alshaal T., Rady A.M.S., El-Sherif A.M.A., Omara A.E.-D., Abd El-Monem A.M., Hafez E.M. (2021). The Integrated Amendment of Sodic-Saline Soils Using Biochar and Plant growth promoting microbesEnhances Maize (*Zea mays* L.) Resilience to Water Salinity. Plants.

[18] Abdel Megeed T.M., Gharib H.S., Hafez E.M., El-Sayed A. (2021). Effect of some plant growth regulators and biostimulants on the productivity of Sakha108 rice plant (*Oryza sativa* L.) under different water stress conditions. Appl. Ecol. Environ. Res..

[19] Hafez E.M., El Hassan W.H.A., Gaafar I.A., Seleiman M.F. (2015). Effect of Gypsum Application and Irrigation Intervals on Clay Saline-Sodic Soil Characterization, Rice Water Use Efficiency, Growth, and Yield. J. Agric. Sci..

[20] Hafez E.M., Alsohim A.S., Farig M., Omara A.-D., Rashwan E., Kamara M.M. (2019). Synergistic Effect of Biochar and Plant growth promoting microbes on Alleviation of Water Deficit in Rice Plants under Salt-Affected Soil. Agronomy.

[21] Kamara M.M., Rehan M., Mohamed A.M., El Mantawy R.F., Kheir A.M.S., Abd El-Moneim D., Safhi F.A., ALshamrani S.M., Hafez E.M., Behiry S.I. (2022). Genetic Potential and Inheritance Patterns of Physiological, Agronomic and Quality Traits in Bread Wheat under Normal and Water Deficit Conditions. Plants.

[22] Isawa T., Sameshima R., Mitsui H., Minamisawa K. (1999). IS1631 occurrence in Bradyrhizobium japonicum highly reiterated sequence-possessing strains with high copy numbers of repeated sequences RS_ and RSß. Appl. Environ. Microbiol..

[23] Harman G.E. (2011). Multi-functional fungal plant symbionts: New tools to enhance plant growth and productivity. New Phytol..

[24] Hafez E.M., Omara A.E.D., Alhumaydhi F.A., El-Esawi M.A. (2021). Minimizing hazard impacts of soil salinity and water stress on wheat plants by soil application of vermicompost and biochar. Physiol. Plant..

[25] Abbasi M.K., Tahir M.M., Azam W., Abbas Z., Rahim N. (2012). Soybean yield and chemical composition in response to phosphorus-potassium nutrition in Kashmir. Agron. J..

[26] Nelson K.A., Motavalli P.P., Stevens W.E., Kendig J.A., Dunn D., Nathan M. (2012). Foliar potassium fertilizer additives affect soybean response and weed control with glyphosate. Int. J. Agron..

[27] Okba S.K., Mazrou Y., Elmenofy H.M., Ezzat A., Salama A.-M. (2021). New Insights of Potassium Sources Impacts as Foliar Application on ‘Canino’ Apricot Fruit Yield, Fruit Anatomy, Quality and Storability. Plants.

[28] Aytaç Z., Gülbandılar A., Kürkçüoğlu M. (2022). Humic Acid Improves Plant Yield, Antimicrobial Activity and Essential Oil Composition of Oregano (*Origanum vulgare* L. subsp. hirtum (Link.) Ietswaart). Agronomy.

[29] Turan M., Ekinci M., Kul R., Kocaman A., Argin S., Zhirkova A.M., Perminova I.V., Yildirim E. (2022). Foliar Applications of Humic Substances Together with Fe/Nano Fe to Increase the Iron Content and Growth Parameters of Spinach (*Spinacia oleracea* L.). Agronomy.

[30] El-Hashash E.F., Abou El-Enin M.M., Abd El-Mageed T.A., Attia M.A.E.-H., El-Saadony M.T., El-Tarabily K.A., Shaaban A. (2022). Bread Wheat Productivity in Response to Humic Acid Supply and Supplementary Irrigation Mode in Three Northwestern Coastal Sites of Egypt. Agronomy.

[31] Wang N., Zhang Q., Han W., Bai C., Hou B., Liu Y., Wang S. (2022). Chemical Characteristics of Dark-Brown Humic-Like Substances Formed from the Abiotic Condensation of Maillard Precursors with Different Glycine Concentrations. Agronomy.

[32] Koushik Ch., Debarati B., Har N.M., Kuldeepsingh K. (2016). External potassium (K^+^) application improves salinity tolerance by promoting Na^+^ exclusion, K^+^ accumulation and osmotic adjustment in contrasting peanut cultivars. Plant Physiol. Biochem..

[33] Ma Y., Dias M.C., Freitas H. (2020). Drought and Salinity Stress Responses and Microbe-Induced Tolerance in Plants. Front. Plant Sci..

[34] Bharti N., Barnawal D., Awasthi A., Yadav A., Kalra A. (2014). Plant growth promoting microbes alleviate salinity induced negative effects on growth, oil content and physiological status in Mentha arvensis. Acta Physiol. Plant..

[35] Dodd I.C., Perez-Alfocea F. (2012). Microbial amelioration of crop salinity stress. J. Exp. Bot..

[36] Abdel-Rahman H.M., Zaghloul R.A., Enas A., Hassan H.R.A., Salem A.A. (2021). New Strains of Plant growth promoting microbesin Combinations with Humic Acid to Enhance Squash Growth under Saline Stress. Egypt. J. Soil. Sci..

[37] Ehsan T., Pichu R., Glenn K. (2010). High concentrations of Na^+^ and Cl^–^ ions in soil solution have simultaneous detrimental effects on growth of faba bean under salinity stress. J. Exp. Bot..

[38] Misra S., Dixit V.K., Khan M.H., Kumar M.S., Dviwedi G., Yadav S., Lehri A., Chauhan P.S. (2017). Exploitation of agro-climatic environment for selection of 1-aminocyclopropane-1-carboxylic acid (ACC) deaminase producing salt tolerant indigenous plant growth promoting microbes. Microbiol. Res..

[39] Li H.Q., Jiang X.W. (2017). Inoculation with plant growth-promoting bacteria (PGPB) improves salt tolerance of maize seedling. Russ. J. Plant Physiol..

[40] Ma W., Penrose D.M., Glick B.R. (2002). Strategies used by rhizobia to lower plant ethylene levels and increase nodulation. Can. J. Microbiol..

[41] Rahi A.A., Anjum M.A., Iqbal Mirza J., Ahmad Ali S., Marfo T.D., Fahad S., Danish S., Datta R. (2021). Yield Enhancement and Better Micronutrients Uptake in Tomato Fruit through Potassium Humate Combined with Micronutrients Mixture. Agriculture.

[42] Hasanuzzaman M., Parvin K., Anee T.I., Masud A.A.C., Nowroz F., Hasanuzzaman M., Nahar K. (2022). Salt Stress Responses and Tolerance in Soybean. Plant Stress Physiology—Perspectives in Agriculture.

[43] Araújo S.S., Beebe S., Crespi M., Delbreil B., González E.M., Gruber V., Lejeune-Henaut I., Link W., Monteros M.J., Prats E. (2015). Abiotic stress responses in legumes: Strategies used to cope with environmental challenges. Crit. Rev. Plant Sci..

[44] Pagano M.C., Miransari M., Miransari M. (2016). The importance of soybean production worldwide. Abiotic and Biotic Stresses in Soybean Production.

[45] Han H.S., Lee K.D. (2005). Physiological responses of soybean-inoculation of Bradyrhizobium japonicum with PGPMS in saline soil conditions. Res. J. Agric. Biol. Sci..

[46] Masciarelli O., Llanes A., Luna V. (2014). A new PGPMS co-inoculated with *Bradyrhizobium japonicum* enhances soybean nodulation. Microbiol. Res..

[47] Egamberdieva D., Wirth S., Jabborova D., Räsänen L.A., Liao H. (2017). Coordination between Bradyrhizobium and Pseudomonas alleviates salt stress in soybean through altering root system architecture. J. Plant Interact..

[48] El-Nahrawy S., Elbagory M., Omara A.E.-D. (2020). Biocompatibility effect of Bradyrhizobium japonicum and Trichoderma strains on growth, nodulation and physiological traits of soybean (Glycine max l.) under water deficit conditions. J. Adv. Microbiol..

[49] Kurutas E.B. (2016). The importance of antioxidants which play the role in cellular response against oxidative/nitrosative stress: Current state. Nutr. J..

[50] Gholami H., Samavat S., Ardebili Z.O. (2013). The alleviating effects of humic substances on photosynthesis and yield of Plantago ovate in salinity conditions, International Research. J. Appl. Basic Sci..

[51] Ferreira R.R., Fornazier R.F., Vitória A.P., Lea P.J., Azevedo R.A. (2002). Changes in antioxidant enzyme activities in soybean under cadmium stress. J. Plant Nutr..

[52] Alam M.Z., Carpenter-Boggs L., Hoque A., Ahammed G.J. (2020). Effect of soil amendments on antioxidant activity and photo-synthetic pigments in pea crops grown in arsenic contaminated soil. Heliyon.

[53] Ahmad R., Hussain S., Anjum M.A., Khalid M.F., Saqib M., Zakir I., Hassan A., Fahad S., Ahmad S., Hasanuzzaman M., Hakeem K.R., Nahar K., Alharby H.F. (2019). Oxidative stress and antioxidant defense mechanisms in plants under salt stress. Plant Abiotic Stress Tolerance: Agronomic, Molecular and Biotechnological Approaches.

[54] Yan J.M., Smith M.D., Glick B.R., Liang Y. (2014). Effects of ACC deaminase containing rhizobacteria on plant growth and expression of Toc GTPases in tomato (*Solanum lycopersicum*) under salt stress. Botany.

[55] Sokol N.W., Slessarev E., Marschmann G.L., Nicolas A., Blazewicz S.J., Brodie E.L., Firestone M.K., Foley M.M., Hestrin R., Hungate B.A. (2022). Life and death in the soil microbiome: How ecological processes influence biogeochemistry. Nat. Rev. Microbiol..

[56] Yaseen R., Aziz O., Saleem M.H., Riaz M., Zafar-ul-Hye M., Rehman M., Ali S., Rizwan M., Nasser Alyemeni M., El-Serehy H.A. (2020). Ameliorating the drought stress for wheat growth through application of ACC-deaminase containing rhizobacteria along with biogas slurry. Sustainability.

[57] Chapman H.D., Pratt P.F. (1962). Methods of Analysis for Soils, Plants and Waters. Soil Sci..

[58] Lichtenthaler H.K. (1987). Chlorophylls and carotenoids: Pigments of photosynthetic biomembranes. Methods in Enzymology.

[59] Velikova V., Yordanov I., Edreva A. (2000). Oxidative stress and some antioxidant systems in acid rain-treated bean plants: Protective role of exogenous polyamines. Plant Sci..

[60] Du Z., Bramlage W.J. (1992). Modified thiobarbituric acid assay for measuring lipid oxidation in sugar-rich plant tissue extracts. J. Agric. Food Chem..

[61] Aebi H. (1984). Catalase in vitro. Methods in Enzymology.

[62] Beauchamp C., Fridovich I. (1971). Superoxide dismutase: Improved assays and an assay applicable to acrylamide gels. Anal. Biochem..

[63] Vetter J.L., Steinberg M.P., Nelson A.I. (1958). Enzyme assay, quantitative determination of peroxidase in sweet corn. J. Agric. Food Chem..

[64] Weatherley P.E. (1950). Studies in the water relations of the cotton plant. I. The field measurement of water deficits in leaves. New Phytol..

[65] Bajji M., Kinet J.-M., Lutts S. (2002). The use of the electrolyte leakage method for assessing cell membrane stability as a water stress tolerance test in durum wheat. Plant Growth Regul..

[66] Bates L.S., Waldren R.P., Teare I.D. (1973). Rapid determination of free proline for water-stress studies. Plant Soil.

[67] Misra P.S., Mertz E.T., Glover D.V. (1975). Studies on corn proteins. VIII. Free amino acid content of opaque-2 double mutants. Cereal Chem..

[68] Bradford M.M. (1976). A rapid and sensitive method for the quantitation of microgram quantities of protein utilizing the principle of protein-dye binding. Anal. Biochem..

[69] Hendrix D.L. (1993). Rapid extraction and analysis of nonstructural carbohydrates in plant tissues. Crop Sci..

[70] A.O.A.C (2005). Official Methods of Analysis of AOAC International.

[71] Sadasivam S., Manickam A. (2010). Biochemical Methods.

