# Supplementary material for: Potassium Humate and Plant Growth-Promoting Microbes Jointly Mitigate Water Deficit Stress in Soybean Cultivated in Salt-Affected Soil
Source: Plants (Basel). 2022 Nov 8;11(22):3016. doi: 10.3390/plants11223016 (PMC9698740; doi:10.3390/plants11223016)
Supplement: Supplementary file 1 [file plants-1959794-supplementary.pdf › plants-1959794-supplementary.pdf]

Table S1: Pearson correlation between the measured parameters of soybean grown in salt-affected soil and exposed to two water regimes (WS: water deficit regime and WW: well-watered regime) and treated with plant growth-promoting microbes (PGPMs) and potassium humate (K-humate)

Water regime	0.00																											
Treatments	0.00	0.00																										
Root length	0.13	0.70**	0.58*																									
Nodule mass	0.05	0.76**	0.52*	0.98**																								
Chl a	-0.15	0.75**	0.64**	0.87**	0.90**																							
Chl b	0.21	0.70**	0.66**	0.93**	0.91**	0.91**																						
Carotenoids	0.18	0.70**	0.66**	0.94**	0.92**	0.92**	0.99**																					
Photosyn. rate	0.12	0.75**	0.63**	0.94**	0.94**	0.95**	0.98**	0.98**																				
RWC	0.10	0.72**	0.67**	0.95**	0.94**	0.95**	0.98**	0.98**	0.99**																			
Stomatal Cond.	0.12	0.73**	0.67**	0.95**	0.94**	0.96**	0.98**	0.98**	0.99**	1.00**																		
Na^+	0.09	-0.75**	-0.64**	-0.91**	-0.94**	-0.99**	-0.92**	-0.93**	-0.96**	-0.97**	-0.97**																	
K^+	0.11	0.71**	0.68**	0.94**	0.94**	0.96**	0.98**	0.98**	0.99**	1.00**	1.00**	-0.97**																
K^+/Na^+	0.04	0.72**	0.66**	0.95**	0.93**	0.96**	0.98**	0.98**	0.99**	0.99**	0.99**	-0.96**	0.99**															
CAT	0.01	-0.52*	-0.82**	-0.88**	-0.86**	-0.93**	-0.93**	-0.93**	-0.94**	-0.94**	-0.94**	0.92**	-0.94**	-0.95**														
POD	0.36	-0.67**	-0.59**	-0.77**	-0.83**	-0.94**	-0.77**	-0.80**	-0.82**	-0.84**	-0.84**	0.93**	-0.85**	-0.84**	0.82**													
SOD	0.08	-0.74**	-0.65**	-0.91**	-0.93**	-0.98**	-0.93**	-0.93**	-0.95**	-0.97**	-0.97**	0.99**	-0.97**	-0.96**	0.91**	0.94**												
MDA	0.07	-0.73**	-0.67**	-0.93**	-0.94**	-0.98**	-0.95**	-0.94**	-0.97**	-0.98**	-0.98**	0.99**	-0.98**	-0.98**	0.94**	0.91**	0.99**											
H_2O_2	0.09	0.55*	-0.73*	-0.12	-0.02	-0.08	-0.12	-0.14	-0.08	-0.12	-0.10	0.05	-0.12	-0.15	0.38	0.05	0.07	0.11										
Electrolyte leakage	0.08	-0.76**	-0.64**	-0.92**	-0.94**	-0.99**	-0.94**	-0.94**	-0.97**	-0.98**	-0.98**	0.99**	-0.98**	-0.97**	0.92**	0.93**	0.99**	0.99**	0.06									
# pods	0.19	0.67**	0.69**	0.93**	0.90**	0.91**	0.99**	0.99**	0.98**	0.98**	0.98**	-0.91**	0.98**	0.98**	-0.94**	-0.76**	-0.92**	-0.94**	-0.19	-0.93**								
100-seed weight	0.48	0.64**	0.59**	0.87**	0.83**	0.76**	0.94**	0.92**	0.90**	0.91**	0.91**	-0.81**	0.91**	0.88**	-0.81**	-0.59**	-0.82**	-0.83**	-0.05	-0.82**	0.92**							
Seed yield	0.26	0.72**	0.62**	0.91**	0.89**	0.91**	0.98**	0.98**	0.96**	0.97**	0.97**	-0.92**	0.97**	0.95**	-0.89**	-0.78**	-0.92**	-0.93**	-0.05	-0.92**	0.97**	0.94**						
Seed protein	0.10	0.76**	0.64**	0.94**	0.94**	0.96**	0.98**	0.98**	1.00**	0.99**	0.99**	-0.97**	0.99**	0.99**	-0.93**	-0.84**	-0.96**	-0.98**	-0.06	-0.98**	0.97**	0.91**	0.96**					
Seed carbs	0.16	0.77**	0.61**	0.94**	0.94**	0.94**	0.98**	0.97**	0.99**	0.99**	0.99**	-0.96**	0.99**	0.98**	-0.91**	-0.81**	-0.95**	-0.97**	-0.02	-0.97**	0.97**	0.93**	0.97**	0.99**				
Leaf proline	0.04	-0.77**	-0.64**	-0.92**	-0.93**	-0.99**	-0.96**	-0.95**	-0.98**	-0.98**	-0.98**	0.99**	-0.98**	-0.98**	0.94**	0.90**	0.98**	0.99**	0.06	0.99**	-0.95**	-0.84**	-0.94**	-0.99**	-0.98**			
Leaf protein	0.13	0.75**	0.63**	0.93**	0.93**	0.95**	0.99**	0.99**	1.00**	0.99**	0.99**	-0.95**	0.99**	0.99**	-0.93**	-0.81**	-0.94**	-0.96**	-0.08	-0.96**	0.99**	0.91**	0.97**	0.99**	0.99**	-0.98**		
Leaf amino acids	0.12	-0.73**	-0.67**	-0.92**	-0.92**	-0.99**	-0.94**	-0.94**	-0.96**	-0.97**	-0.97**	0.98**	-0.97**	-0.98**	0.94**	0.93**	0.99**	1.00**	0.13	0.99**	-0.93**	-0.80**	-0.92**	-0.97**	-0.95**	0.99**	-0.96**	
Leaf total soluble sugars	0.04	0.73**	0.65**	0.94**	0.92**	0.95**	0.98**	0.97**	0.98**	0.99**	0.98**	-0.95**	0.98**	1.00**	-0.94**	-0.84**	-0.95**	-0.97**	-0.14	-0.96**	0.98**	0.87**	0.95**	0.98**	0.97**	-0.98**	0.98**	-0.98**
	Year	Water regime	Treatments	Root length	Nodule mass	Chl a	Chl b	Carotenoids	Photosyn. rate	RWC	Stomatal Cond.	Na^+	K^+	K^+/Na^+	CAT	POD	SOD	MDA	H_2O_2	Electrolyte leakage	# pods	100-seed weight	Seed yield	Seed protein	Seed carbs	Leaf proline	Leaf protein	Leaf amino acids

**. Correlation is significant at the 0.01 level (2-tailed).
*. Correlation is significant at the 0.05 level (2-tailed).
